# Resistance analysis of genotype 3 hepatitis C virus indicates subtypes inherently resistant to nonstructural protein 5A inhibitors

**DOI:** 10.1002/hep.29837

**Published:** 2018-04-27

**Authors:** David Smith, Andrea Magri, David Bonsall, Camilla L.C. Ip, Amy Trebes, Anthony Brown, Palo Piazza, Rory Bowden, Dung Nguyen, M. Azim Ansari, Peter Simmonds, Eleanor Barnes, STOP‐HCV Consortium

**Affiliations:** ^1^ Nuffield Department of Medicine and the Oxford NIHR BRC University of Oxford Oxford UK; ^2^ Oxford Martin School University of Oxford Oxford UK; ^3^ Wellcome Trust Centre for Human Genetics University of Oxford Oxford UK

## Abstract

Hepatitis C virus (HCV) genotype (gt) 3 is highly prevalent globally, with non‐gt3a subtypes common in Southeast Asia. Resistance‐associated substitutions (RASs) have been shown to play a role in treatment failure. However, the role of RASs in gt3 is not well understood. We report the prevalence of RASs in a cohort of direct‐acting antiviral treatment‐naive, gt3‐infected patients, including those with rarer subtypes, and evaluate the effect of these RASs on direct‐acting antivirals *in vitro*. Baseline samples from 496 gt3 patients enrolled in the BOSON clinical trial were analyzed by next‐generation sequencing after probe‐based enrichment for HCV. Whole viral genomes were analyzed for the presence of RASs to approved direct‐acting antivirals. The resistance phenotype of RASs in combination with daclatasvir, velpatasvir, pibrentasvir, elbasvir, and sofosbuvir was measured using the S52 ΔN gt3a replicon model. The nonstructural protein 5A A30K and Y93H substitutions were the most common at 8.9% (n = 44) and 12.3% (n = 61), respectively, and showed a 10‐fold and 11‐fold increase in 50% effect concentration for daclatasvir compared to the unmodified replicon. Paired RASs (A30K + L31M and A30K + Y93H) were identified in 18 patients (9 of each pair); these combinations were shown to be highly resistant to daclatasvir, velpatasvir, elbasvir, and pibrentasvir. The A30K + L31M combination was found in all gt3b and gt3g samples. *Conclusion:* Our study reveals high frequencies of RASs to nonstructural protein 5A inhibitors in gt3 HCV; the paired A30K + L31M substitutions occur in all patients with gt3b and gt3g virus, and *in vitro* analysis suggests that these subtypes may be inherently resistant to all approved nonstructural protein 5A inhibitors for gt3 HCV. (Hepatology 2018).

**Abbreviations**
DAA: direct‐acting antiviral
EC50: 50% effect concentration
gt: genotype
HCV: hepatitis C virus
NS: nonstructural protein
RAS: resistance‐associated substitution
RLU: relative light unit
SVR: sustained viral response
WT: wild type

Hepatitis C virus (HCV) infection is a global health problem, with 71 million infected people worldwide and 1.75 million new infections each year according to recent World Health Organization estimates.^1^ The recent development of direct‐acting antivirals (DAAs) has led to a dramatic increase in sustained viral response (SVR) rates, with many studies reporting >90% SVR rates.^2^, ^3^, ^4^, ^5^ Despite this dramatic increase in effectiveness of DAA treatment for chronic HCV infection, the treatment of genotype 3 (gt3) infection has shown lower SVR rates compared to other genotypes, especially in patients with cirrhosis.^6^, ^7^ This contrasts with the treatment of gt3‐infected individuals with interferon‐based therapy in whom SVRs were consistently higher than in those with gt1.^8^, ^9^ More recently, pan‐genotypic regimens have been developed that very effectively target HCV gt3.^10^, ^11^, ^12^, ^13^, ^14^

The reasons for the reduced efficacy of some interferon‐free DAA therapies against gt3 infection remain unclear. Host genetics such as interferon lambda 4 loci, which is associated with clinical and biological outcomes for HCV,^15^, ^16^ could be a contributing factor. Infection with gt3 HCV has also been associated with clinical phenotypes that may affect response to DAA therapy, including hepatic steatosis, increased rates of liver fibrosis,^17^ and increased chance of progression to hepatocellular carcinoma,^18^, ^19^ each of which has been linked to poor outcomes after DAA therapy^20^; and this may help to explain the reduced efficacy of DAAs in gt3. Presence of resistance‐associated substations (RASs) in viral sequences could be another factor contributing to lower SVR rates in gt3. For instance, the Y93H substitution has a high prevalence in gt3 sequences and has been shown in some studies to be associated with lower SVR rates, especially in patients with cirrhosis.^7^, ^21^

The current recommendation for the treatment of gt3 infection from the European Association for the Study of the Liver (2016) is a combination of one of the nonstructural protein 5A (NS5A) inhibitors, daclatasvir or velpatasvir, with the NS5B polymerase inhibitor sofosbuvir.^22^ The American Association for the Study of Liver Diseases (2017) recommends one of the following combinations depending on previous treatment experience and the presence or absence of cirrhosis and hepatic decompensation: glecaprevir/pibrentasvir, velpatasvir/sofosbuvir, voxilaprevir/velpatasvir/sofosbuvir, or grazoprevir/elbasvir/sofosbuvir.^23^ The Asian‐Pacific Association for the Study of the Liver recommendations are now outdated (2016) and advocate the use of either sofosbuvir with ribavirin or the combination of daclatasvir and sofosbuvir ± ribavirin depending on treatment experience and liver disease state.^24^

Viral variants carrying RASs have been reported in clinical trials for all current DAAs,^25^, ^26^, ^27^ many of which have been characterized *in vitro* using replicon‐based or virus‐based resistance assays. For non‐gt2 HCV variants, the role of RASs has been typically evaluated using subgenomic replicons where the structural protein region has been replaced with a luciferase reporter that allows direct quantitation of replication.^28^ The transient‐replication assay, based on viral RNA transfection followed by short‐term monitoring of viral replication through the reporter gene, is the preferred method for RAS testing because of the reduced chance of adaptive mutations and a higher throughput than models which use stable replicon cell lines expressing viral RNA.^29^ The gt3a replicon S52/SG‐Feo, used in a transient‐replication assay, was recently improved by removing the neomycin resistance gene (ΔN).^29^ We used this replicon with a modified Huh 7.5 cell line expressing a stable, high level of the SEC14‐L2 gene^29^ that enhances HCV replication^30^ to assess the phenotype of RASs in a gt3a background in a transient‐replication model.

In this study we investigated the frequencies of potential RASs in a large (n = 496) gt3 cohort (prior to sofosbuvir‐based treatment regimens in the BOSON clinical study) using a probe‐based sequence capture approach for next‐generation sequencing to generate full‐length HCV genomes^31^ and bioinformatics tools to detect viral variants at frequencies of <1%.^32^, ^33^ The phenotypic effect of RASs was evaluated *in vitro* both individually and in combination using the gt3a replicon system, and their potential roles in treatment failure were evaluated.

## Materials and Methods

### Subjects and Samples

Samples were obtained from patients enrolled in the BOSON study^34^ before treatment commenced. All patients were DAA treatment‐naive and received sofosbuvir and ribavirin for 16 or 24 weeks or sofosbuvir, ribavirin, and pegylated interferon for 12 weeks. All patients provided written informed consent before undertaking any study‐related procedures. The BOSON study protocol was approved by each institution's review board or ethics committee before study initiation. The study was conducted in accordance with the International Conference on Harmonization Good Clinical Practice Guidelines^35^ and the Declaration of Helsinki.

### Library Construction and Enrichment and Next‐Generation Sequencing

RNA was extracted from 500 μL of plasma using the NucliSENS easyMAG system (bioMérieux) into 30 μL of water, of which 5 μL was processed with the NEBNext Ultra Directional RNA Library Prep Kit for Illumina (New England Biolabs) with published modifications to the manufacturer's protocol.^36^ A 500‐ng aliquot of the pooled library was enriched using the xGen Lockdown protocol (Rapid Protocol for DNA Probe Hybridization and Target Capture Using an Illumina TruSeq Library [v1.0]; Integrated DNA Technologies) with a comprehensive panel of HCV‐specific, 120‐nucleotide DNA oligonucleotide probes (Integrated DNA Technologies), designed using a published algorithm.^31^ The enriched library was sequenced using Illumina MiSeq v2 chemistry to produce paired 150‐bp reads.

### Bioinformatics

Reads were demultiplexed, 150‐bp paired‐end reads were trimmed (QUASR; Cutadapt), and host‐derived sequences were removed (Bowtie). Reads of viral origin were selected (blastn) for *de novo* assembly (Vicuna), read mapping (MOSAIK), genome annotation (VFAT), and interpretation of variants (genewise2, Vphaser, Vprofiler). Further detail of the techniques used can be found in Bonsall et al.^31^

### Drugs

Daclatasvir, velpatasvir, and elbasvir were purchased from MedchemExpress; sofosbuvir was purchased from Selleckchem; and pibrentasvir was purchased from MedKoo.

### HCV Replicon Plasmid

The HCV Gt3a replicon S52‐ΔN (L/GDD_ΔN) plasmid contains the HCV internal ribosome entry site driving the translation of the firefly luciferase gene and the encephalomyocarditis virus internal ribosome entry site responsible for the synthesis of the HCV polyprotein (NS3‐NS5B) (Supporting Fig. S1). This replicon has been recently improved by removing the neomycin resistance gene.^29^ The plasmid DNA was linearized using *XbaI* (New England Biolabs) and *in vitro* transcribed as described.^37^, ^38^

### Generation of HCV Mutant Replicons

RASs were introduced into the HCV gt3a replicon using the QuikChange II XL Site‐Directed Mutagenesis Kit (Agilent Technologies). Positions in this article refer to H77 consensus; for a full list of positions in S52‐ΔN and H77, see Supporting Table S1. All reactions were transformed into Top10 bacteria (Life Technologies), and for each mutation three clones were isolated and sequenced to confirm the viral sequence.

### Cell Culture and HCV Transfection

Huh7.5 cells overexpressing SEC14L2 protein (Huh7.5‐SEC14L2) were generated as reported by our lab.^29^ In brief, Huh7.5 cells were transduced with a lentiviral vector to overexpress SEC142 protein. After puromycin selection, single‐cell clones were isolated and characterized as described.^29^ Cells were maintained in Dulbecco's modified Eagle's medium supplemented with 10% fetal calf serum, 10 mM 4‐(2‐hydroxyethyl)‐1‐piperazine ethanesulfonic acid buffer, 2 mM L‐glutamine, 100 U/mL penicillin, 100 μg/mL streptomycin, and 0.1 M nonessential amino acids. RNA transfection was performed by electroporating 4 to 5 × 10^6^ cells for each reaction with 2 μg of RNA as described.^29^, ^37^

### Evaluation of Viral Fitness

To infer the replicative fitness of RAS‐containing viruses, cells transfected with each construct were resuspended in complete medium at the approximate concentration of 3 × 10^5^ cells/mL and seeded into 96‐well plates, and then luciferase activity (relative light units [RLUs]) was measured after 4, 24, 48, and 72 hours using Bright Glo (Promega) on the GloMax 96 Microplate Luminometer (Promega). The replication capacity of the variant was calculated using the following equation: Replication capacity = (Variant 72‐hour RLU/Variant 4‐hour RLU)/(wild type [WT] 72‐hour RLU/WT 4‐hour RLU). The replication‐defective pSGR‐JFH1/GND replicon, containing a self‐inactivating mutation in the NS5B gene, was used as a negative control.

### Evaluation of the Resistance Phenotype

Huh7.5‐SEC14L2 cells transfected as described above were seeded in the presence of daclatasvir or sofosbuvir over the appropriate concentration range. For the single RASs 3‐fold dilutions were prepared starting at 3 μM for sofosbuvir and 300 pM for daclatasvir. The susceptibility of multiple NS5A RASs was evaluated using 10‐fold dilutions of daclatasvir, velpatasvir, pibrentasvir, and elbasvir starting at 100 μM. Each experiment included at least two replicates and was performed at least twice. The 50% effect concentration (EC50) values were calculated using nonlinear regression on GraphPad Prism 7.00 for Mac (GraphPad Software, La Jolla, CA) (Supporting Figs. S2‐S7).

## Results

### Review of the Literature for RAS Definitions

**Figure 1:**
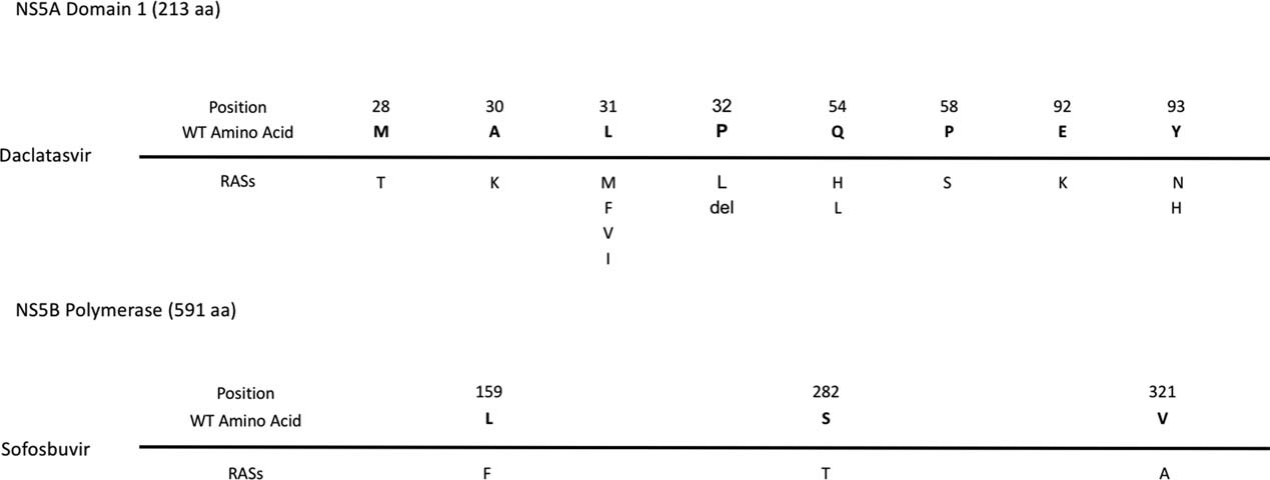
Summary of daclatasvir and sofosbuvir RASs identified in the literature. Key RAS positions for daclatasvir and sofosbuvir are shown (not to scale) along the NS5A domain 1 and NS5B (polymerase) proteins, respectively, labeled according to the H77 gt1 HCV reference protein sequence. The WT amino acid for gt3 is shown above. and the relevant RASs are indicated below. Data taken from Nelson et al.,^7^ Leroy V et al.,^21^ Lontok E et al.,^25^ and Itakura J et al.^39^ Abbreviation: aa, amino acid.

A search through the currently available literature for gt3 RASs to daclatasvir and sofosbuvir revealed five sites in the NS5A region associated with daclatasvir resistance and three sites in the NS5B region associated with sofosbuvir resistance. Sites 32, 54, and 92 in NS5A were also included as they have been shown to be associated with resistance in other genotypes; however, their effect in gt3 is unclear. The RASs at these sites are summarized in Fig. 1.^7^, ^21^, ^25^, ^39^

### Whole‐Genome Sequencing of HCV to Identify RAS

Whole‐genome HCV sequences were obtained for 518 of the 530 gt3 patients enrolled in the study. The viral variant data were analyzed for the prevalence of RASs. If there was insufficient sequence depth for variant calling at any of the 11 RAS codons, the sequence was discarded. Complete sets of viral variant data for the 11 RAS sites in NS5A and NS5B were obtained for 496 gt3 patients.

### Prevalence of RASs to Daclatasvir and Sofosbuvir in a gt3 Population

**Figure 2:**
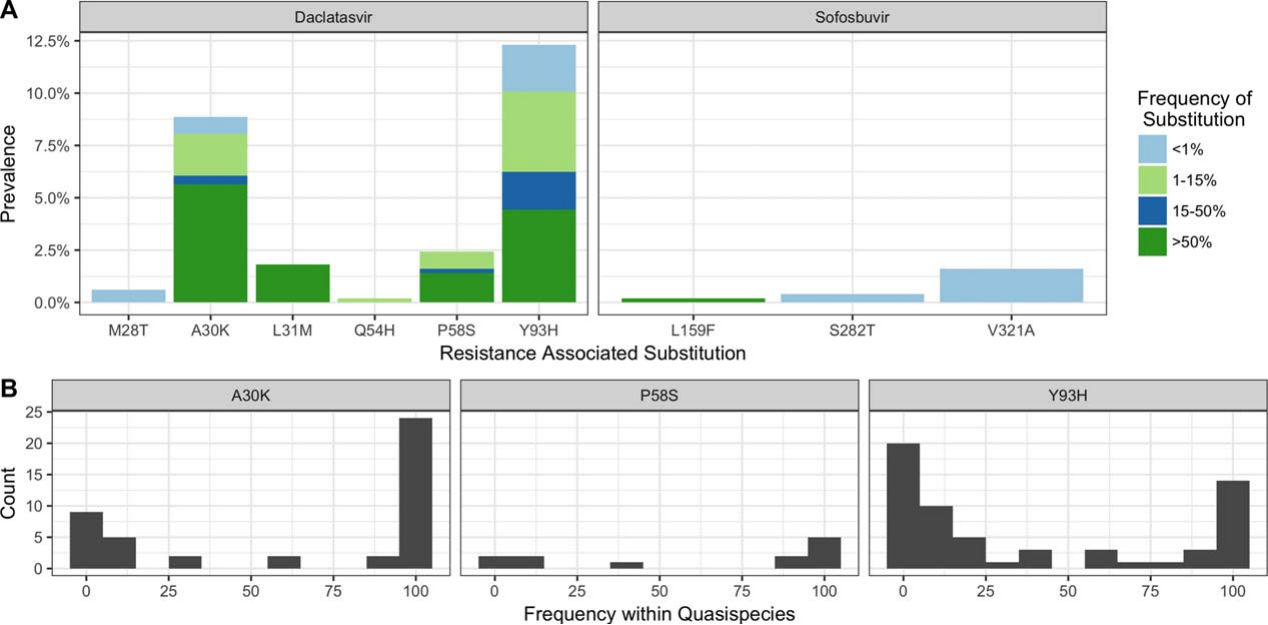
Prevalence of RASs to daclatasvir and sofosbuvir. (A) The prevalence of individual daclatasvir and sofosbuvir RASs, categorized by frequency within viral quasi‐species. (B) Histograms with a bin size of 10% showing the distribution of substitution frequency within individual patient viral quasi‐species for the daclatasvir RASs A30K, P58S, and Y93H.

HCV sequences were analyzed for the presence of RASs to daclatasvir and sofosbuvir. We observed six daclatasvir RASs M28T, A30K*, L31M, Q54H*, P58S*, and Y93H in NS5A, (*corresponding to Q30K, T54H, and H58S in gt1a; Supporting Table S1) and three sofosbuvir RASs L159F, S282T, and V321A in NS5B. The frequencies of A30K and Y93H RASs were particularly high (Fig. 2A), being detected in 8.9% (n = 44) and 12.3% (n = 61) of samples, respectively. We also found L31M in 1.8% (n = 9), P58S in 2.4% (n = 12), M28T in 0.6% (n = 3), and Q54H in 0.2% (n = 1) of the samples. The prevalence of amino acid substitutions not associated with resistance at these sites can be found in Supporting Table S2.

Overall, 130 individual RASs to daclatasvir were identified in 110 samples; of those, 14% (n = 18) were detected in <1% of the quasi‐species, 26% (n = 34) were low‐frequency variants (1%‐15% of viral quasi‐species), 9% (n = 12) were minor variants (present in 15%‐50% of quasi‐species), and 51% (n = 66) were major variants (present in >50% of quasi‐species). A much smaller percentage of samples carried RASs to sofosbuvir (2.2%, n = 11). The L159F substitution was found in 0.2% (n = 1) of samples at 100% of the quasi‐species, the S282T in 0.4% (n = 2), and the V321A in 1.6% (n = 8) of the samples at <1% of the quasi‐species. The majority of RASs were detected as either low‐frequency variants or majority variants, with a minority of variants being detected at frequencies between 15% and 85% (Fig. 2B).

### Phenotypic Evaluation of Individual RASs to Daclatasvir and Sofosbuvir using a gt3 Replicon System

**Figure 3:**
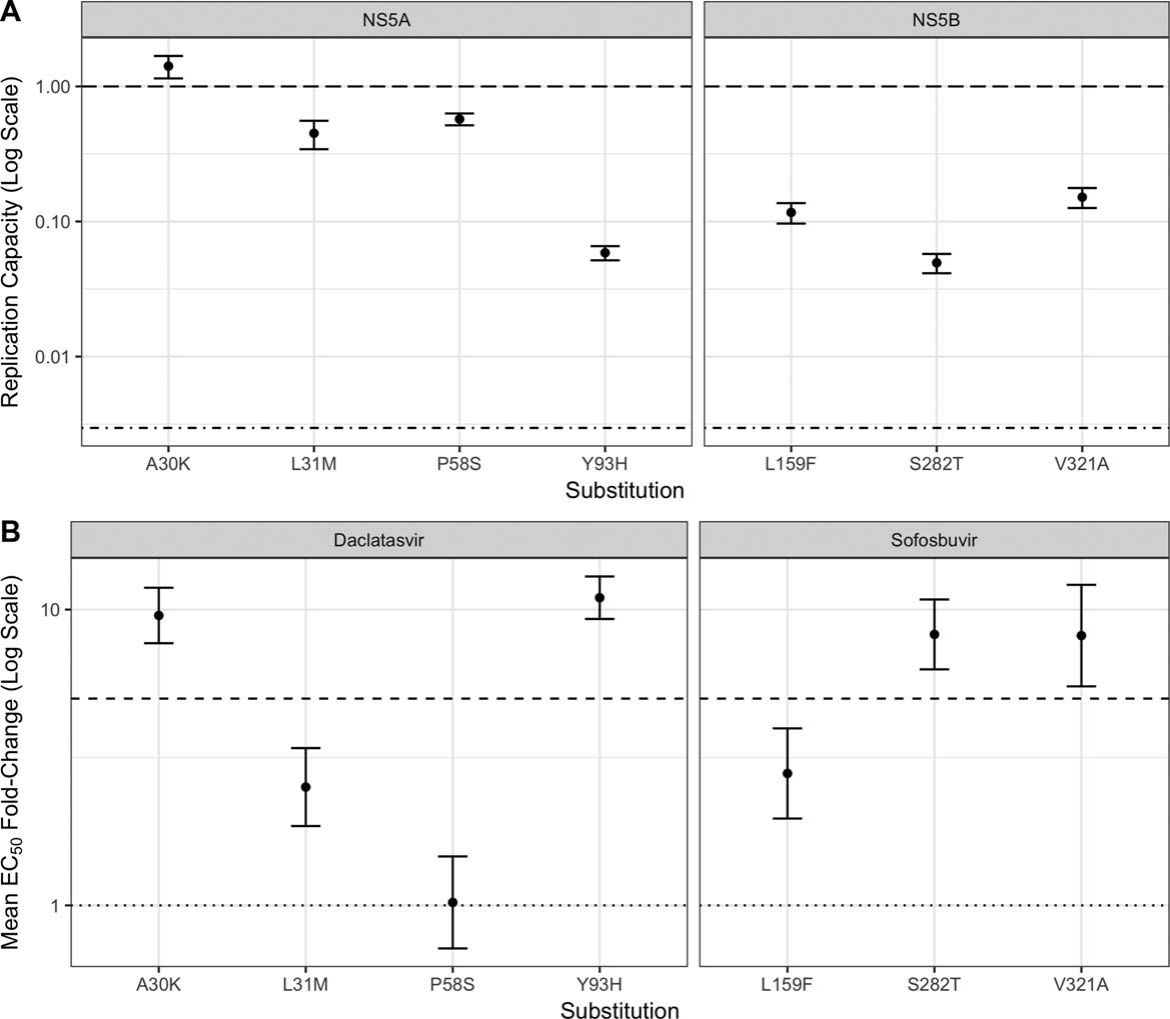
(A) Replication capacity of S52 ΔN replicon carrying candidate RASs. The top dashed and bottom dotted lines indicate the replication capacity of the unmodified WT S52 ΔN replicon and the pSGR‐JFH1/GND replicon, respectively. (B) Fold changes in resistance (based on EC50 measurements) for daclatasvir or sofosbuvir against S52 ΔN replicons carrying candidate RASs. The dotted line indicates WT susceptibility; the dashed line indicates a 5‐fold increase in EC50 when compared to the WT S52 ΔN replicon. Error bars show standard error of the mean calculated from at least two experiments.

We selected all NS5A RASs detected in >1% of the samples (A30K, L31M, P58S, Y93H) and all three detected NS5B RASs (L159F, S282T, and V321A) for phenotypic characterization. To assess the effects of each individual RAS to daclatasvir and sofosbuvir, we generated mutant replicons harboring the single substitution. Initially, we investigated whether individual RASs might affect viral fitness in the absence of DAAs. Interestingly, compared to WT, A30K exhibited an increase of ∼0.5‐fold in replication capacity and P58S and L31M showed a reduction of ∼3‐fold in replication, whereas Y93H, L159F, S282T, and V321A showed substantially impaired replication by ∼13‐fold, ∼7‐fold, ∼16‐fold, and ∼5‐fold, respectively, relative to WT levels (Fig. 3A).

To assess the variants' susceptibility to daclatasvir or sofosbuvir, the EC50 of each RAS‐carrying replicon to the relevant drug was measured using a dose–response scale (Fig. 3B). For daclatasvir, the NS5A substitutions A30K and Y93H showed a >5‐fold increase in EC50, while the L31M substitution only showed an increase of ∼2‐fold in EC50 and the P58S substitution showed no significant change in EC50 when compared to WT. For sofosbuvir, the NS5B substitutions S282T and V321A showed a >5‐fold increase in EC50 and the L159F substitution showed an increase of ∼2‐fold in EC50 compared to WT.

### Frequencies and Associated Resistance Phenotypes of RAS Combinations to NS5A Inhibitors in gt3 Populations

**Figure 4:**
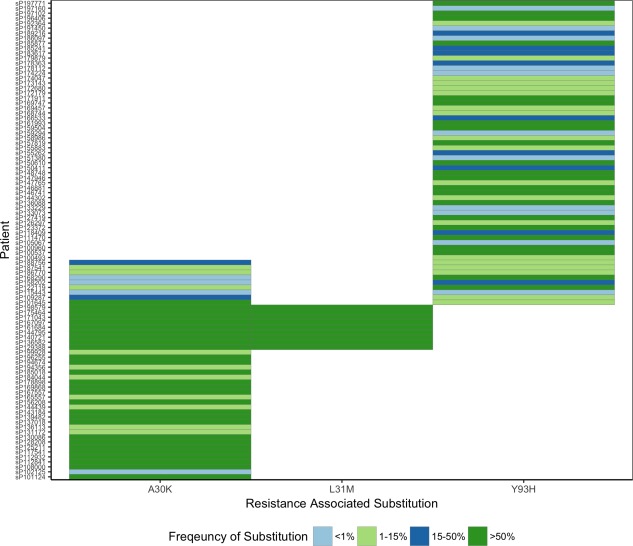
Prevalence of RAS combinations to daclatasvir. Each column represents a RAS site, and each row is a patient. The frequency of the substitution within the viral quasi‐species is shown in each block.

To investigate the effects of multiple RASs on the DAA resistance phenotype, we scanned pretreatment sequences from the cohort for combinations of RASs which individually showed at least a 2‐fold increase in EC50 in our assay. A total of 9 patients had the combination of A30K + Y93H variant and 9 patients had the combination of A30K + L31M in the NS5A protein (Fig. 4). No NS5B RASs were detected in combination. In samples carrying the A30K + L31M combination, both RASs were present as the majority variant. In all of the samples with the A30K + Y93H combinations at least one of the RASs was detected at a frequency of <50% (Fig. 4).

**Figure 5:**
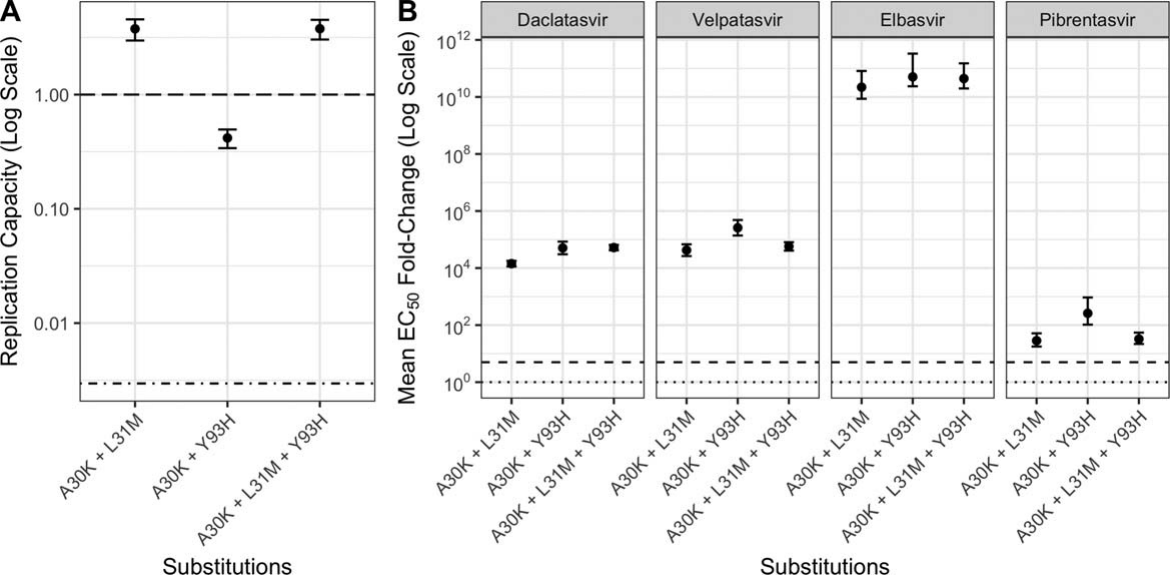
(A) Replication capacity of S52 ΔN replicon carrying candidate NS5A RAS combinations. The top dashed and bottom dotted lines indicate the replication capacity of the unmodified WT S52 ΔN replicon and the pSGR‐JFH1/GND replicon, respectively. (B) EC50 fold change for daclatasvir, velpatasvir, elbasvir, or pibrentasvir against S52 ΔN replicon carrying candidate RAS combinations. The dotted line indicates no change in EC50 compared to WT; the dashed line indicates a 5‐fold increase in EC50 when compared to the WT S52 ΔN replicon.

The effect of paired RASs on replication capacity and NS5A inhibitor susceptibility was first evaluated using daclatasvir in our *in vitro* system. A30K + L31M had a replication capacity 3‐fold greater than WT, while the combination A30K + Y93H had a 1‐fold lower replication capacity than WT (Fig. 5A). Both A30K + L31M and A30K + Y93H combinations showed a >10,000‐fold increase in EC50 (Fig. 5B).

We also generated a triple variant combination of A30K + L31M + Y93H RASs. This was not found among the pretreatment sequences from the cohort but may represent a possible additional synergistic interaction. A30K + L31M + Y93H showed a 3‐fold increase in replication capacity and a >10,000‐fold increase in the EC50 value for daclatasvir when compared to WT (Fig. 5B).

Due to the large increase in daclatasvir EC50 values for the combined RASs, we next evaluated these RAS against the NS5A inhibitors velpatasvir, pibrentasvir, and elbasvir, which have recently been approved for the treatment of gt3 HCV infection. The double combinations A30K + L31M and A30K + Y93H and the triple combination A30K + L31M + Y93H also showed a highly resistant phenotype to both velpatasvir (>10,000‐fold increase in EC50) and elbasvir (>100,000,000‐fold increase in EC50). Resistance to pibrentasvir was also observed but at lower levels (>20‐fold increase in EC50). The relatively higher fold change in EC50 with elbasvir may be explained by the increased susceptibility of the WT replicon to this drug (Supporting Fig. S8).

### The Prevalence of RASs Within HCV gt3 Subtypes

**Figure 6:**
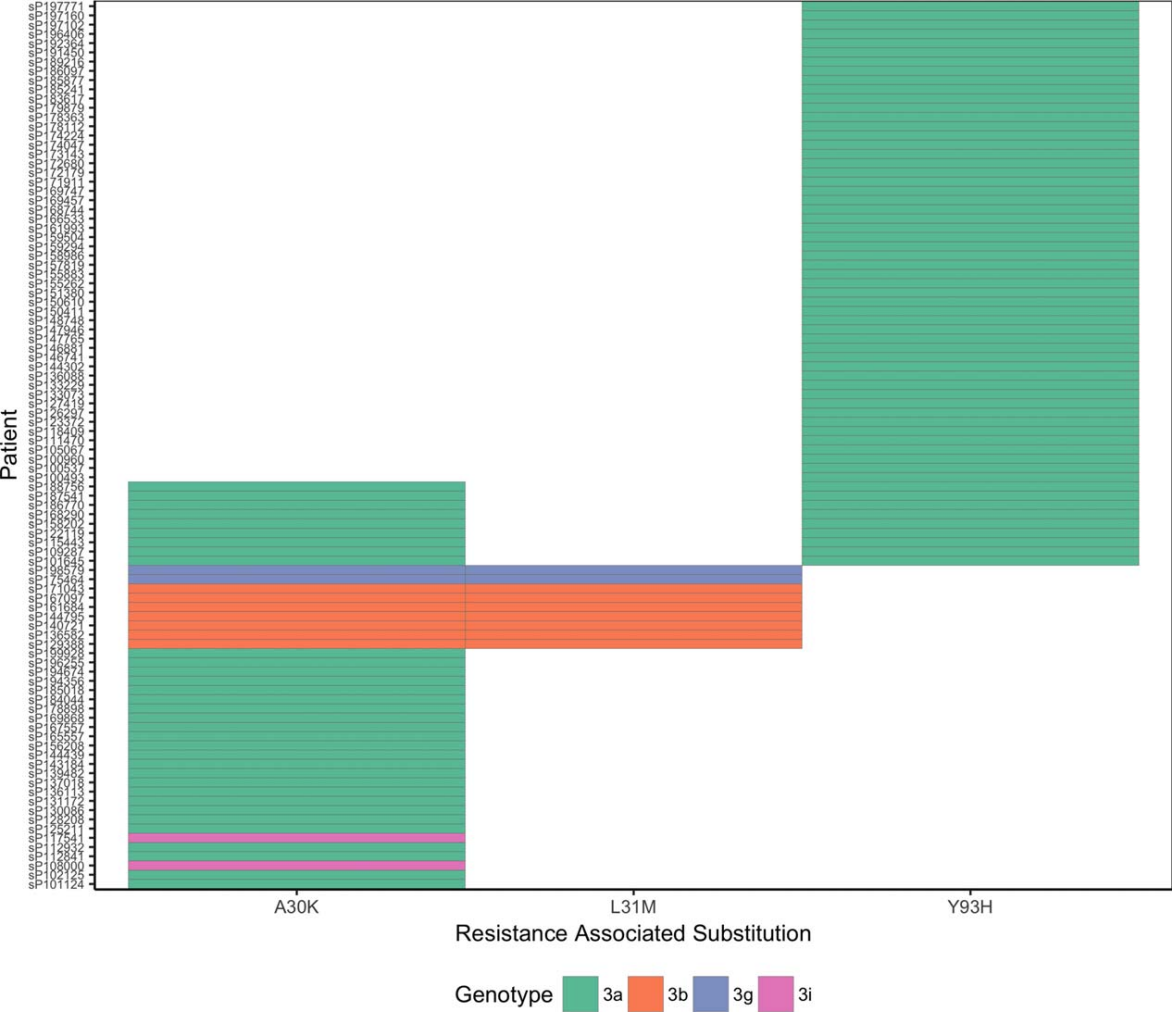
The distribution of RASs within gt3 subtypes. Each column represents an individual RAS, and each row is a patient; each block is colored according to the gt3 subtype of the virus.

**Figure 7:**
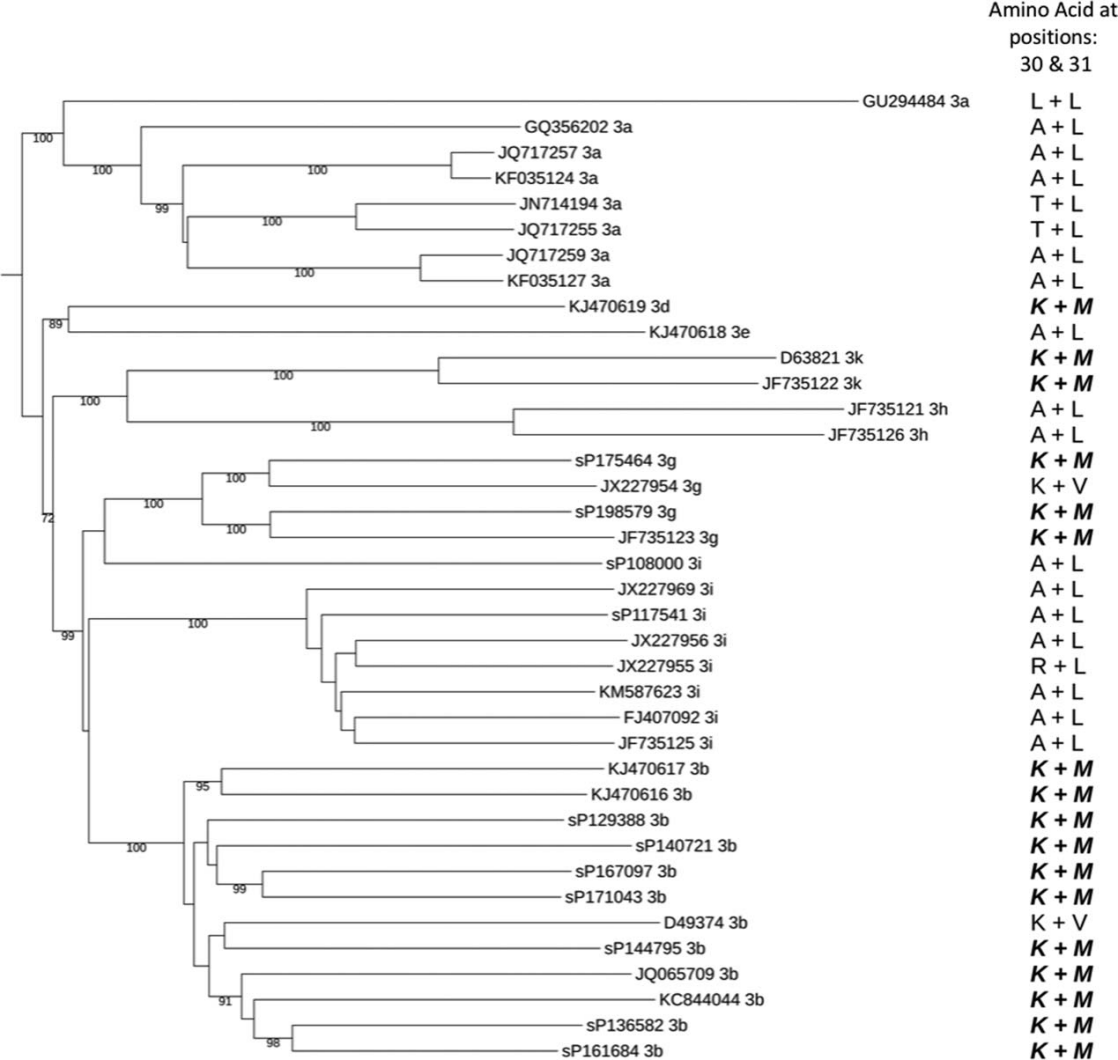
Whole‐genome phylogeny of gt3 subtypes labeled with NS5A position 30 and 31 amino acids. A neighbor‐joining, midpoint rooted phylogenetic tree was estimated using the whole‐genome sequence of the gt3b, gt3g, and gt3i subtypes from our cohort and the gt3a, gt3d, gt3e, gt3k, gt3h, gt3i, gt3g, and gt3b sequences downloaded from the National Center for Biotechnology Information database and annotated with the NS5A position 30 and 31 amino acids. The A30K + L31M RASs are in bold and italics.

The distribution of RASs conferring high‐level resistance to NS5A inhibitors was strongly associated with the genetic background of the virus (Fig. 6). The L31M RAS was only observed in non‐gt3a subtypes (gt3b, seven samples; gt3g, two samples). Additionally, all nine samples that carried the L31M RAS also carried the A30K RAS. All other RASs were restricted to the gt3a subtype except for the A30K RAS found in two gt3i‐infected patients. Of the 9 patients carrying gt3b and gt3g viruses, eight were of Asian origin and one was of unknown origin. To further evaluate the global distribution of the A30K + L31M combination in the gt3 subtypes, we analyzed 27 additional gt3 full‐genome consensus sequences from the National Center for Biotechnology Information together with the 11 non‐gt3a sequences from our cohort for the presence of the A30K + L31M substitutions (Fig. 7). Interestingly, the A30K + L31M substitutions were found in all but two gt3b and gt3g sequences as well as the one gt3d and the two gt3k sequences, suggesting that the A30K + L31M substitutions are likely to be the WT residues for these subtypes.

## Discussion

This study reports the prevalence of RASs in a large cohort of DAA treatment‐naive, gt3‐infected patients using next‐generation virus whole‐genome sequencing. From these data, a list of RASs was compiled for further characterization using the S52 gt3a replicon. We show that the frequency of RASs to daclatasvir or sofosbuvir in gt3 was 22% and 2%, respectively, comparable to those described recently by Chen et al.^40^ (27.6% and 3.9%) and Patiño‐Galindo et al.,^41^ although both studies used consensus sequences obtained from publicly available databases. In these studies substitutions representing deviations from the consensus sequence were considered to be potential RASs and were based on smaller sample sizes for gt3 (Chen et al. had a sample size of 48, and Patiño‐Galindo et al. used sequences of individual genes with sample sizes of 117 NS3, 226 NS5A, and 81 NS5B). These data highlight a need for more study into gt3 RASs to allow clearer definitions.

Next‐generation sequencing techniques such as the ve‐seq^31^ method, used in this study, allow the detection of very low‐frequency viral variants, 0.5%‐1%^32^; this sensitivity is much better than the Sanger capillary‐based population sequencing used in the majority of studies of RASs in which minor variants at a frequency lower than ∼20% cannot be resolved unless individual clones are sequenced, which is time‐consuming and labor‐intensive.^42^ For example, in our study 40% of the occurrences of gt3 NS5A RASs had variant frequencies of <15% that would have been undetectable by older methodologies.

We report that in gt3 the individual RASs A30K and Y93H provide modest resistance to daclatasvir and that the combinations of A30K + L31M, A30K + Y93H, and A30K + L31M + Y93H provide a dramatic increase in resistance to velpatasvir, daclatasvir, elbasvir, and pibrentasvir. These data agree with Hernandez et al.,^43^ showing a similar phenotype for both A30K + L31M and A30K + Y93H using a JFH1‐gt3a NS5A chimera replicon when treated with daclatasvir. Additionally, we report that L31M and P58S show little or no evidence of resistance to daclatasvir in our gt3a replicon system. We also present evidence that S282T and V321A substitutions in NS5B provide low levels of resistance to sofosbuvir in our assay.

The relationship between the occurrence of RASs and clinical resistance to treatment is additionally influenced by the impact on viral fitness of substitutions that depart from the consensus sequence. Clearly, RASs, however effective they may be at reducing viral susceptibility, may be of no clinical relevance if the mutant viruses are unable to replicate *in vivo*. On this basis, the A30K + L31M combination of substitutions should be viewed with substantial concern clinically as they have been observed in patients, confer both a large increase in EC50 when compared to WT, and substantially enhance viral replication competence, a surrogate measure of basic fitness, albeit in a cell culture–based replicon format. Unexpectedly, this combination was only detected in gt3b and gt3g samples and suggests that its occurrence in gt3a may therefore be associated with an undocumented fitness cost or incompatibility not replicated in *in vitro* assays. The preferential distribution of this RAS combination in non‐gt3a subtypes, typically found in South and Southeast Asia,^44^ suggests that these gt3 subtypes may be naturally more resistant to NS5A inhibitors; but this association remains to be tested clinically. Recent published data has also suggested that gt 1l and 4r may also be naturally resistant to NS5A inhibitors.^45^ In the current cohort, all patients were treated by sofosbuvir +/− ribavirin +/− interferon, and all non‐gt3a‐infected patients achieved sustained viral clearance, except one gt3i‐infected patient, precluding direct testing of NS5A inhibitor resistance on retreatment. Furthermore, the association needs to be retested in a compatible virus genetic background; the high‐resistance phenotype was observed when these RASs were introduced onto the gt3a replicon and may display a different phenotype when present in their native subtype.

Determining at what frequency a RAS becomes clinically relevant remains challenging. European Association for the Study of the Liver guidelines recommend the use of resistance testing for the NS5A region only for patients who are to be retreated having failed on previous all‐oral DAA treatments.^22^ When next‐generation sequencing techniques are used, it is recommended that variants with a frequency of <15% not be considered clinically relevant.^22^ Interestingly, in all of the samples in which the A30K + Y93H substitutions were detected, at least one of the two substitutions was detected with a frequency <15%. Because of the distance between the two sites and the short fragments used in Illumina sequencing, the linkage of these two RASs cannot be confirmed using our data. These data also suggest that the A30K + Y93H combination may not be a fit variant *in vivo* as well as *in vitro*.

Overall, our study shows relatively high frequencies of RASs to the approved NS5A inhibitor daclatasvir in gt3 HCV. We also show that the A30K + L31M RAS combination is the WT for gt3b and gt3g HCV and that this combination provides resistance to all NS5A inhibitors approved for the treatment of gt3 HCV. These findings support the recent recommendation from the American Association for the Study of Liver Diseases^23^ that supports the use of NS3 inhibitors in combination with NS5A inhibitors. Additionally, our findings highlight the need for more study into the efficacy of DAA therapy in less common gt3 subtypes.

## Supporting information

Supporting Information 1
