# Supplementary material for: Resistance analysis of genotype 3 hepatitis C virus indicates subtypes inherently resistant to nonstructural protein 5A inhibitors
Source: Hepatology. 2018 Apr 27;69(5):1861–72. doi: 10.1002/hep.29837 (PMC6492296; doi:10.1002/hep.29837)
Supplement: Supplementary file 1 — Supporting Information 1 [file HEP-69-1861-s001.pdf]

Supplementary Table and Figures

Protein	Position	Position in H77 Polyprotein	Amino Acid in H77	Codon in H77	Position in S52 Polyprotein	Amino acid in S52	Codon in S52	Mutations
NS5A	30	2002	Q	caa	2008	A	gca	K
NS5A	31	2003	L	ctg	2009	L	ctc	M
NS5A	58	2030	H	cac	2036	P	cct	S
NS5A	93	2065	Y	tac	2071	Y	tac	H
NS5B	159	2579	L	ctc	2589	L	ctc	F
NS5B	282	2702	S	agc	2712	S	agt	T
NS5B	321	2741	V	gtg	2751	V	gtg	A

Supplementary Table 1. Positions, amino acids and codons in H77 reference and S52 replicon sequences.

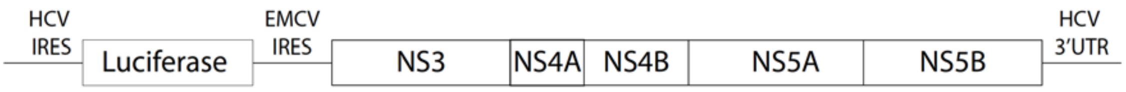

Supplementary Fig 1. Map of S52 ∆N Replicon**.**

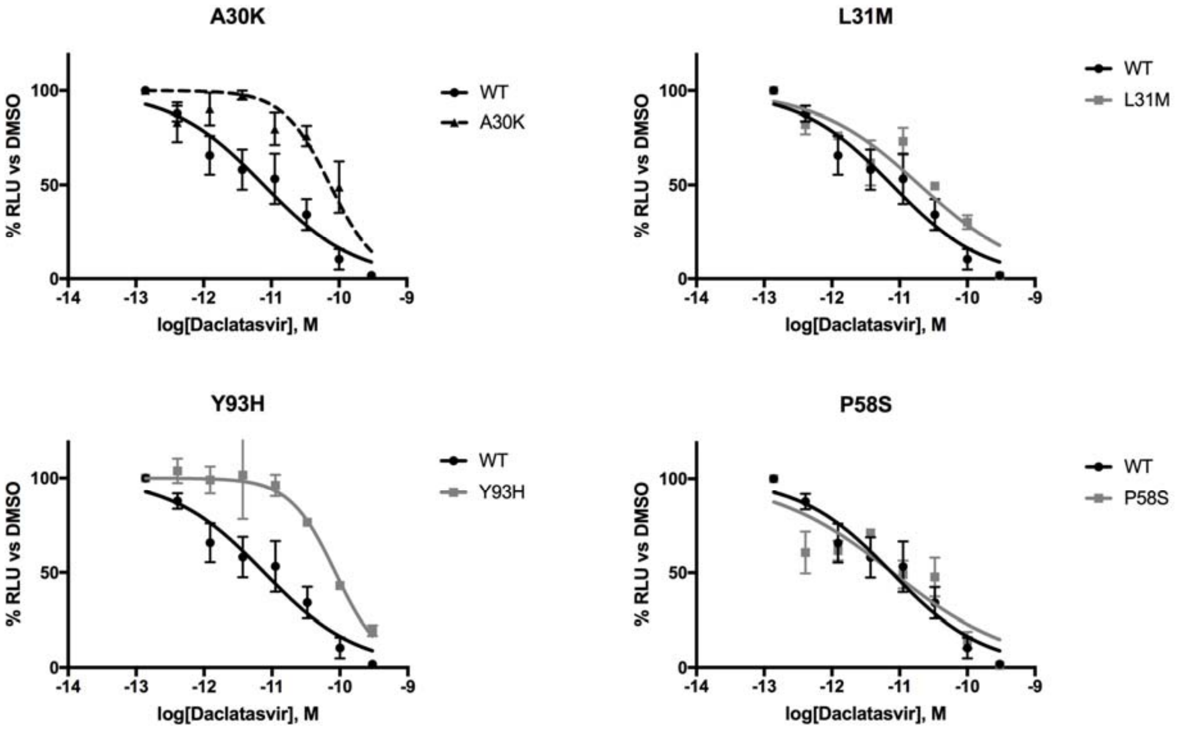

Supplementary Fig 2. Dose-response profiles of individual RASs to daclatasvir.

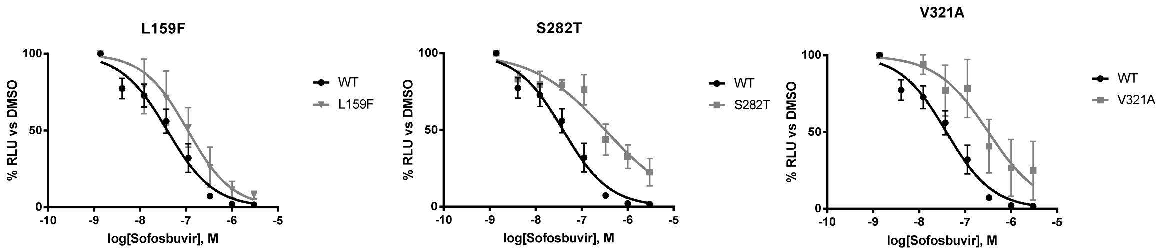

Supplementary Fig 3. Dose-response profiles of individual RASs to sofosbuvir.

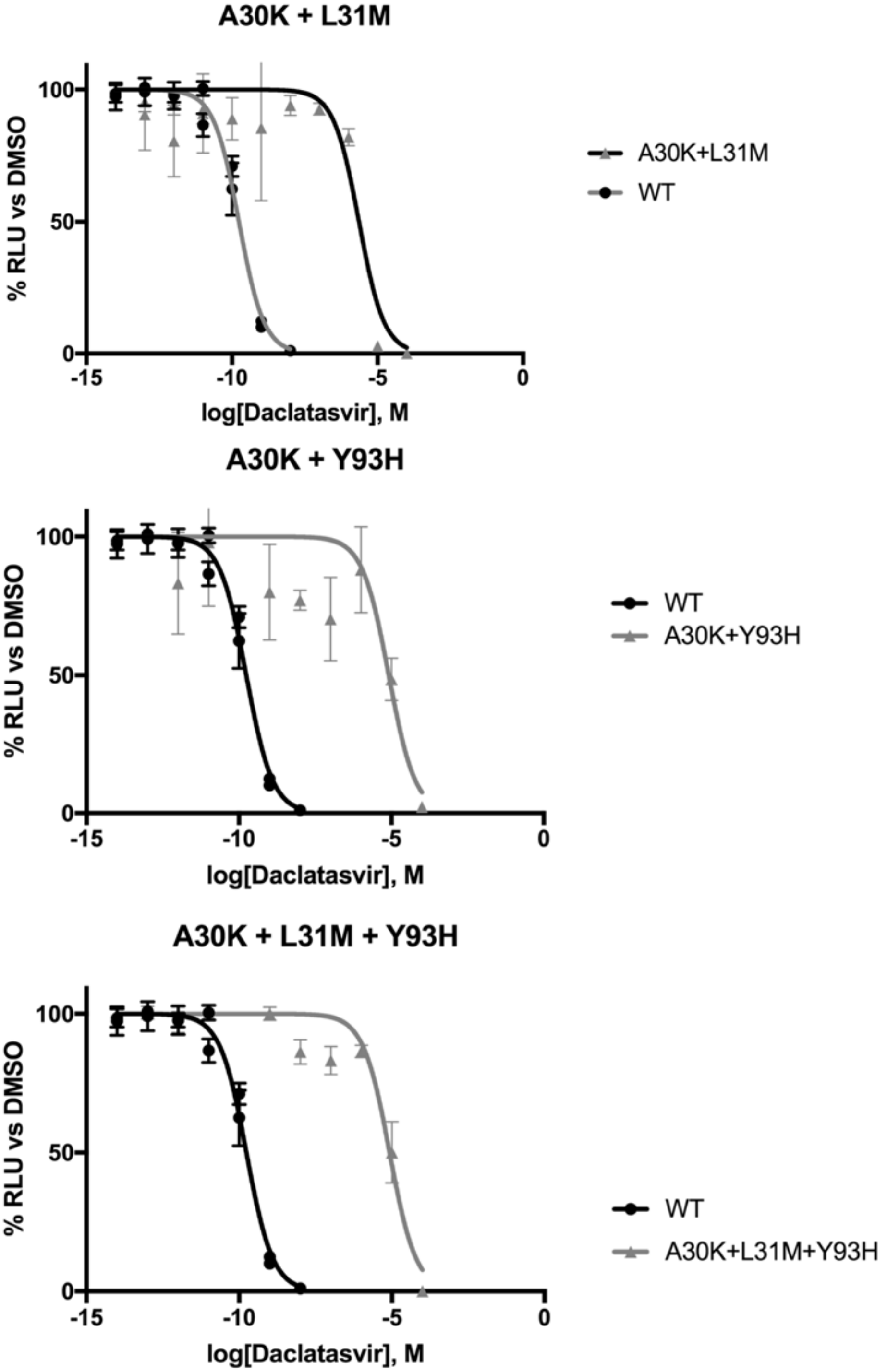

Supplementary Fig 4. Dose-response profiles of RASs combinations to daclatasvir.

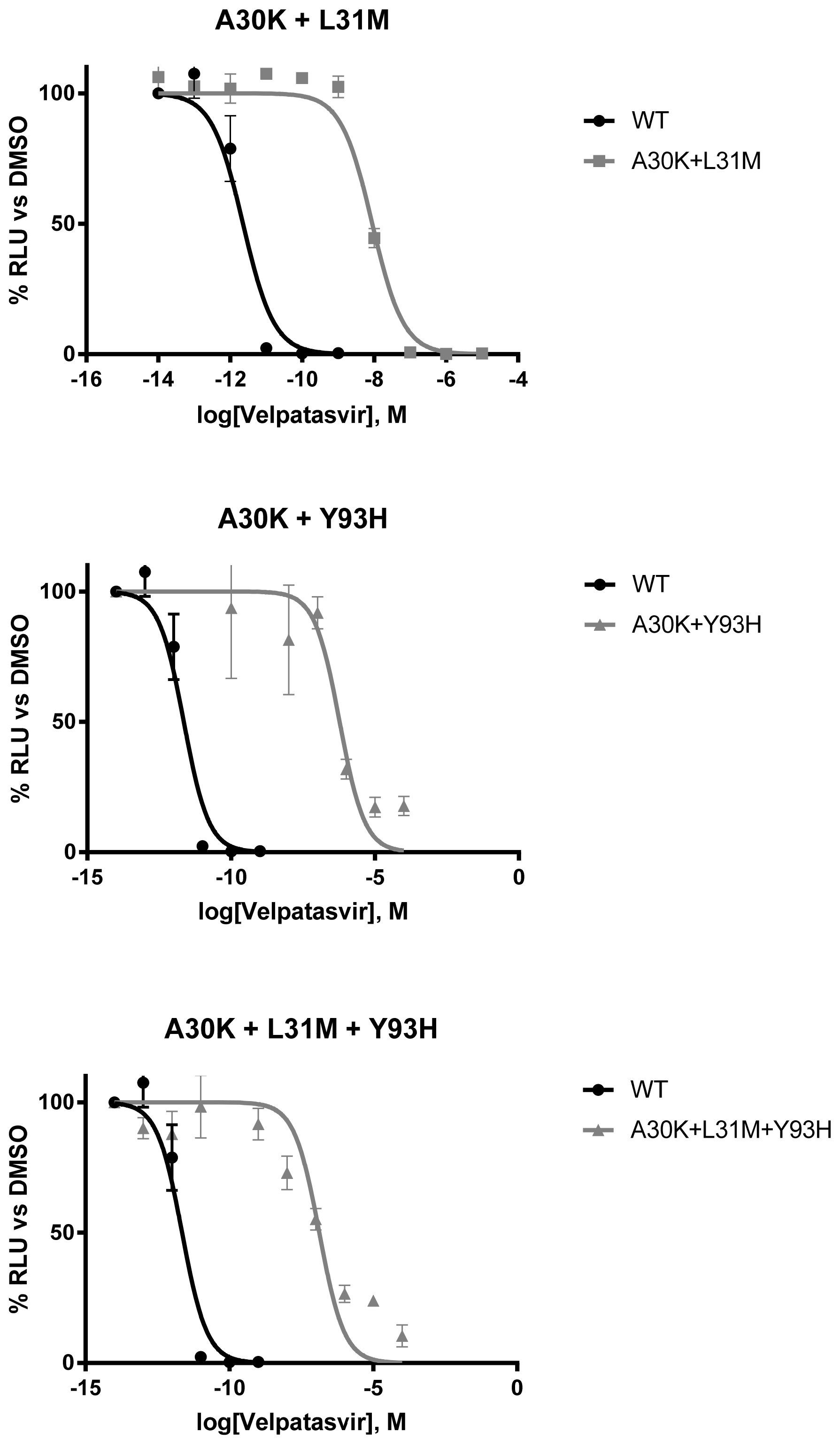

Supplementary Fig 5. Dose-response profiles of RASs combinations to velpatasvir.

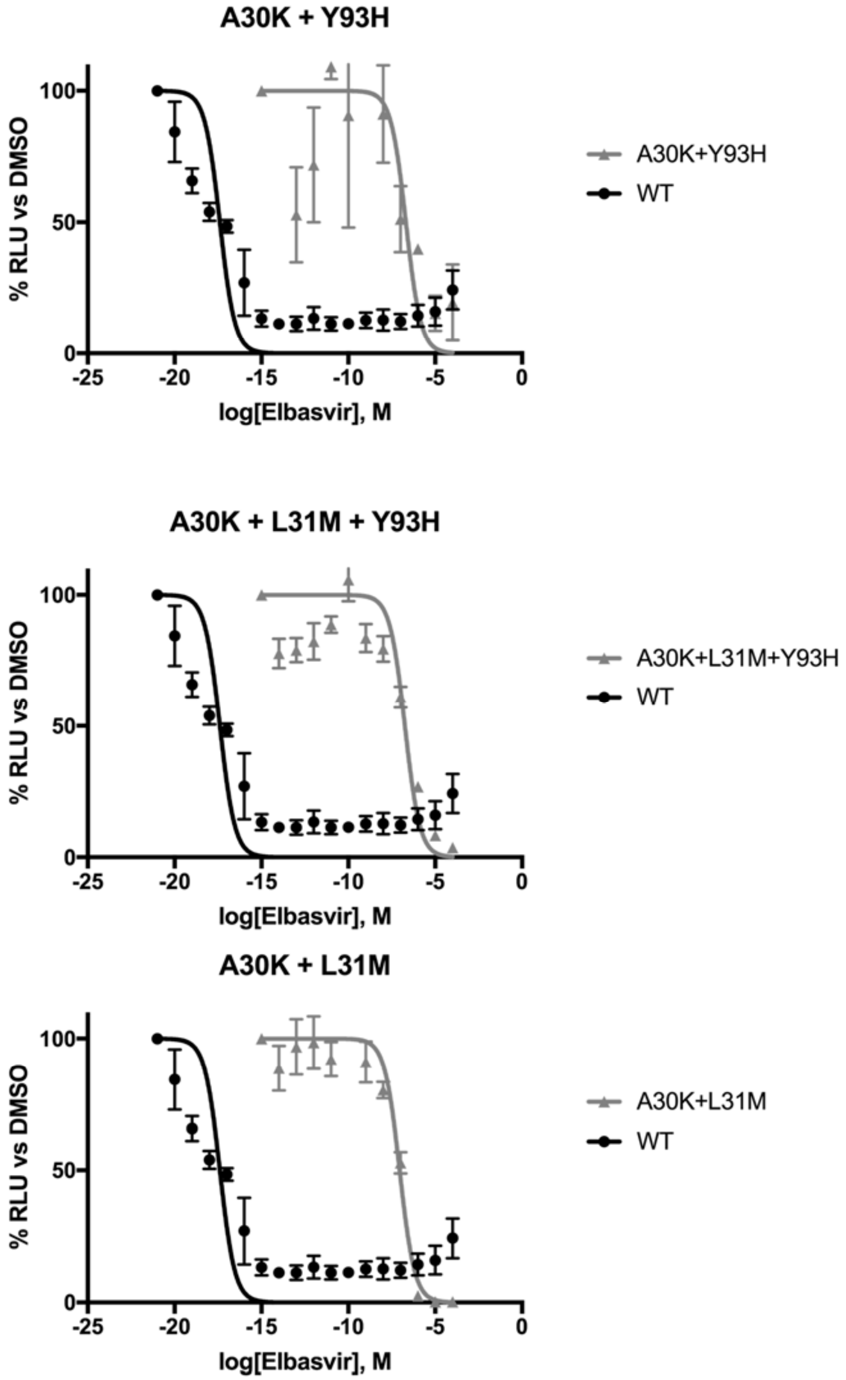

Supplementary Fig 6. Dose-response profiles of RASs combinations to elbasvir.

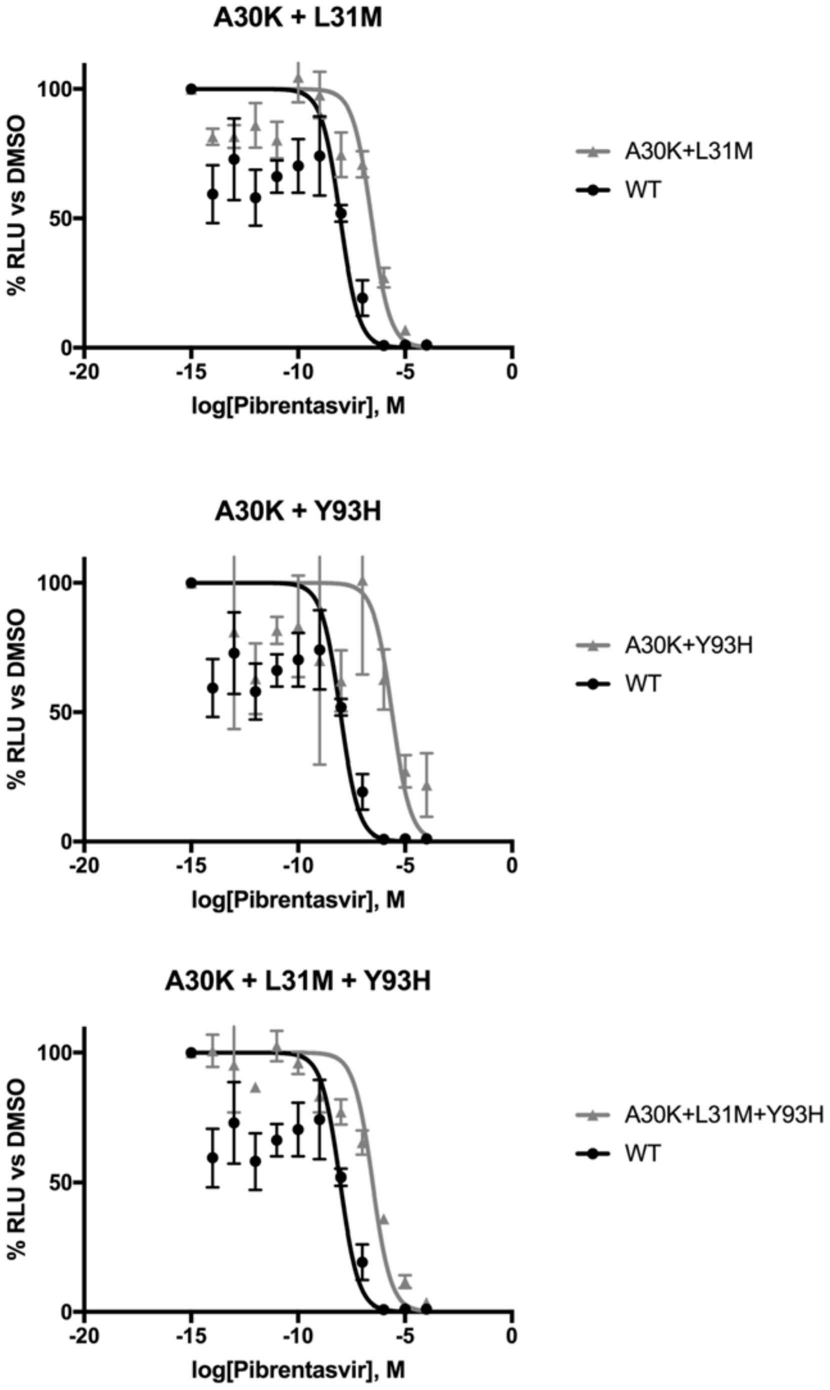

Supplementary Fig 7. Dose-response profiles of RASs combinations to pibrentasvir.

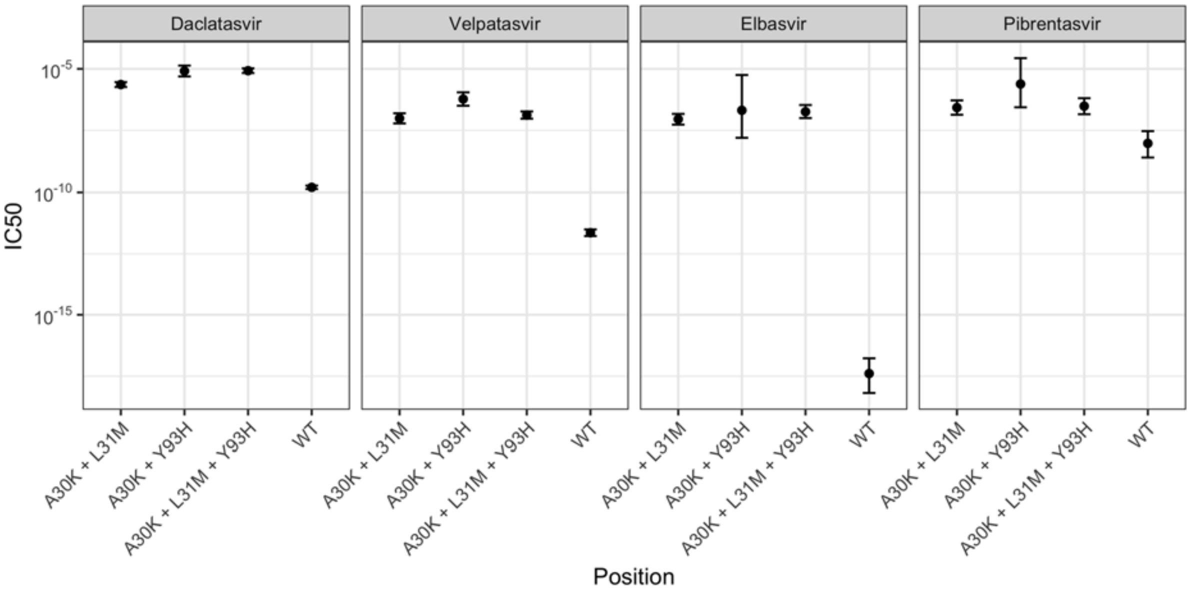

Supplementary Fig 8. EC50 values for daclatasvir, velpatasvir, elbasvir and pibrentasvir against the wild type (WT) gt3a S52 ∆N Replicon and A30K + L31M, A30K + Y93H, A30K + L31M + Y93H variants.

Protein	Position	Amino Acid	Percentage Prevalence (n= 496)			
	Quasispecies Frequency:		<1%	1-15%	15-50%	>50%
NS5A	28	I	0%	0%	0%	0%
NS5A	28	L	0%	0%	0%	0%
NS5A	28	M	0%	0%	0%	100%
NS5A	28	T	1%	0%	0%	0%
NS5A	28	V	0%	1%	0%	1%
NS5A	30	A	1%	2%	2%	91%
NS5A	30	E	1%	0%	0%	0%
NS5A	30	K	1%	2%	0%	6%
NS5A	30	L	0%	0%	0%	0%
NS5A	30	M	1%	0%	0%	0%
NS5A	30	R	0%	0%	0%	0%
NS5A	30	S	1%	0%	0%	2%
NS5A	30	T	2%	0%	1%	1%
NS5A	30	V	1%	2%	1%	1%
NS5A	31	I	0%	0%	0%	0%
NS5A	31	L	0%	0%	0%	100%
NS5A	31	M	0%	0%	0%	2%
NS5A	31	P	0%	0%	0%	0%
NS5A	31	R	1%	0%	0%	0%
NS5A	32	P	0%	0%	0%	100%
NS5A	32	R	0%	0%	0%	0%
NS5A	54	A	0%	0%	0%	0%
NS5A	54	H	0%	0%	0%	0%
NS5A	54	N	0%	0%	0%	0%
NS5A	54	P	0%	0%	0%	0%
NS5A	54	S	1%	3%	1%	93%
NS5A	54	T	2%	1%	1%	8%
NS5A	54	W	0%	0%	0%	0%
NS5A	58	A	0%	1%	0%	1%
NS5A	58	G	0%	0%	0%	0%
NS5A	58	H	0%	0%	0%	0%
NS5A	58	P	0%	1%	0%	98%
NS5A	58	R	0%	0%	0%	1%
NS5A	58	S	0%	1%	0%	1%
NS5A	58	T	0%	0%	0%	0%
NS5A	92	D	0%	0%	0%	0%

NS5A	92	E	0%	0%	0%	100%
NS5A	92	G	0%	0%	0%	0%
NS5A	92	Q	0%	0%	0%	0%
NS5A	93	D	0%	0%	0%	0%
NS5A	93	H	2%	4%	2%	4%
NS5A	93	R	0%	0%	0%	0%
NS5A	93	S	0%	0%	0%	0%
NS5A	93	Y	0%	2%	1%	97%
NS5B	159	F	0%	0%	0%	0%
NS5B	159	I	0%	0%	0%	0%
NS5B	159	L	0%	0%	0%	100%
NS5B	282	G	0%	0%	0%	0%
NS5B	282	S	0%	0%	0%	100%
NS5B	282	T	0%	0%	0%	0%
NS5B	321	A	2%	0%	0%	0%
NS5B	321	F	0%	0%	0%	0%
NS5B	321	I	0%	0%	0%	0%
NS5B	321	L	0%	0%	0%	0%
NS5B	321	M	0%	0%	0%	0%
NS5B	321	V	0%	0%	0%	100%

Supplementary Table 2. Prevalence of amino acids at scanned RAS positions. The prevalence is broken down by the substitution frequency within individual patient viral quasispecies.
